# Long-term prognostic significance of gasping in out-of-hospital cardiac arrest patients undergoing extracorporeal cardiopulmonary resuscitation: a post hoc analysis of a multi-center prospective cohort study

**DOI:** 10.1186/s40560-023-00692-1

**Published:** 2023-10-06

**Authors:** Satoshi Nara, Naofumi Bunya, Hirofumi Ohnishi, Keigo Sawamoto, Shuji Uemura, Nobuaki Kokubu, Mamoru Hase, Eichi Narimatsu, Yasufumi Asai, Yoshio Tahara, Takahiro Atsumi, Ken Nagao, Naoto Morimura, Tetsuya Sakamoto, SAVE-J Study Group

**Affiliations:** 1 Emergency and Critical Care Medical Center, Teine Keijinkai Hospital, Sapporo, Japan; 2 Department of Emergency Medicine, Sapporo Medical University, Sapporo, Japan; 3 Department of Public Health, Sapporo Medical University, Sapporo, Japan; 4 Department of Cardiovascular, Renal and Metabolic Medicine, Sapporo Medical University, Sapporo, Japan; 5 Cardiovascular Center, Sapporo Teishinkai Hospital, Sapporo, Japan; 6 Department of Cardiovascular Emergency, National Cerebral and Cardiovascular Center, Suita, Japan; 7 Department of Emergency Medicine, Seirei Hamamatsu General Hospital, Shizuoka, Japan; 8 Department of Cardiology, Nihon University Hospital, Tokyo, Japan; 9 Department of Emergency Medicine, Teikyo University School of Medicine, Tokyo, Japan

**Keywords:** Gasping, Sign of life, Cardiopulmonary resuscitation, Extracorporeal cardiopulmonary resuscitation, Out-of-hospital cardiac arrest

## Abstract

**Background:**

Gasping during resuscitation has been reported as a favorable factor for out-of-hospital cardiac arrest. We examined whether gasping during resuscitation is independently associated with favorable neurological outcomes in patients with refractory ventricular fibrillation or pulseless ventricular tachycardia (VF/pVT) undergoing extracorporeal cardiopulmonary resuscitation ECPR.

**Methods:**

Data from a 2014 study on advanced cardiac life support for ventricular fibrillation with extracorporeal circulation in Japan (SAVE-J), which examined the efficacy of ECPR for refractory VF/pVT, were analyzed. The primary endpoint was survival with a 6-month favorable neurological outcome in patients who underwent ECPR with or without gasping during resuscitation. Multivariate logistic regression analysis was performed to evaluate the association between gasping and outcomes.

**Results:**

Of the 454 patients included in the SAVE-J study, data from 212 patients were analyzed in this study after excluding those with missing information and those who did not undergo ECPR. Gasping has been observed in 47 patients during resuscitation; 11 (23.4%) had a favorable neurological outcome at 6 months. Multivariate logistic regression analysis showed that gasping during resuscitation was independently associated with a favorable neurological outcome (odds ratio [OR], 10.58 [95% confidence interval (CI) 3.22–34.74]). The adjusted OR for gasping during emergency medical service transport and on arrival at the hospital was 27.44 (95% CI 5.65–133.41).

**Conclusions:**

Gasping during resuscitation is a favorable factor in patients with refractory VF/pVT. Patients with refractory VF/pVT with continuously preserved gasping during EMS transportation to the hospital are expected to have more favorable outcomes.

**Supplementary Information:**

The online version contains supplementary material available at 10.1186/s40560-023-00692-1.

## Background

Despite the accumulating knowledge on cardiopulmonary resuscitation (CPR) over the past decade, survival rates with favorable neurological function to discharge from the hospital remain noticeably low in out-of-hospital cardiac arrest (OHCA), which range from 5% to 10% [1, 2]. The prognosis of OHCA in patients who do not respond to the usual advanced cardiac life support (ACLS) is even poorer [3, 4]. For refractory cardiac arrest, in which spontaneous circulation is not restored despite ACLS, extracorporeal CPR (ECPR) is a rescue therapy expected to contribute to improved outcomes by reducing ischemic brain damage and providing time to identify and treat the underlying reversible causes of cardiac arrest [5, 6]. Although the results of a systematic review, including three recent randomized controlled trials on ECPR, suggest a potential benefit of ECPR, the level of evidence remains low [7–10].

Several reports have shown that signs of life during resuscitation are associated with favorable neurological outcomes for patients with OHCA undergoing ECPR [11–14]. Some studies have reported that the presence of gasping, considered a sign of life, predicts the outcome in patients with OHCA [15–18]. However, previous reports on the usefulness of signs of life, including gasping in ECPR cases, have been limited to reports, wherein the criteria for ECPR were unclear or differed between the periods covered within the study [11, 12]. Thus, whether there is an association between the presence of signs of life and outcomes in patients with refractory OHCA undergoing ECPR remains unclear. A multicenter prospective observational study, SAVE-J, was published in 2014 and provides clear criteria for ECPR implementation for refractory ventricular fibrillation or pulseless ventricular tachycardia (VF/pVT) [19]. Patient information on gasping was collected before and on hospital arrival in the SAVE-J study. We believe that using the SAVE-J data with clear criteria for ECPR implementation to examine the value of gasping as a prognostic factor in ECPR will allow for a more focused discussion of case selection for indicating ECPR with less heterogeneity. The primary aim of this study was to examine whether gasping during resuscitation is independently associated with favorable neurological outcomes in patients with refractory VF/pVT undergoing ECPR.

## Methods

### Study design

Data from the SAVE-J study, which prospectively examined the effect of ECPR on neurological outcomes in patients with refractory OHCA whose initial cardiac rhythm showed VF/pVT between September 8, 2008, and September 30, 2011 [19], were used in this study. Each participating institution was divided into ECPR-performing and non-ECPR-performing institutions, and no randomization was performed. Twenty-two institutions were enrolled in the ECPR group, and 17 institutions in the conventional CPR group. The SAVE-J study collected general prognostic indicators of OHCA and the presence or absence of gasping before and on arrival at the hospital. No information was collected on prehospital advanced airway management in the study. Details of the collective data and resuscitative strategies in the ECPR/CCPR group are available in published data [19].

The SAVE-J study was registered with the University Hospital Medical Information Network Clinical Trials Registry and the Japanese Clinical Trial Registry (registration number: UMIN000001403). This secondary analysis of de-identified data was approved by the Institutional Review Board of Sapporo Medical University Hospital (approval number: 342-163). The requirement for patient consent was waived because of the anonymized data provided in the sub-analysis of the SAVE-J study. The procedures were performed in accordance with the Declaration of Helsinki.

### Patients

Patients enrolled in the SAVE-J study met the following criteria: (1) VF/pVT on the initial cardiac rhythm; (2) cardiac arrest on hospital arrival with or without pre-hospital restoration of spontaneous circulation (ROSC); (3) less than 45 min from the reception of the emergency call or onset of cardiac arrest to hospital arrival; and (4) no ROSC at least during the 15 min after hospital arrival (or after contact with a doctor), even though conventional CPR was performed. Patients excluded in the SAVE-J study were as follows: (a) those aged ≤ 20 years or ≥ 75 years; (b) poor level of activities of daily living before the onset of cardiac arrest; (c) cardiac arrest etiology of non-cardiac origin (e.g., external factors, such as trauma and drug intoxication, primary cerebral disorders, acute aortic dissection diagnosed before the introduction of ECMO, and terminal phase of cancer); (d) core body temperature of < 30 °C; and (e) no informed consent from the individuals representing patients.

### Outcomes

The primary endpoint was survival with a 6-month favorable neurological outcome in patients undergoing ECPR, with or without gasping during resuscitation, evaluated using the cerebral performance category (CPC) [20]. A CPC score of 1–2 and 3–5 was regarded as favorable and unfavorable, respectively. The secondary endpoint was survival with a 6-month favorable neurological outcome in all patients with refractory VF/pVT, regardless of the presence or absence of gasping during resuscitation.

### Statistical analysis

Continuous variables are presented as median (interquartile range) and were compared using the Mann–Whitney *U* test. Categorical variables were compared using Fisher’s exact test. Multivariate logistic regression analysis using stepwise forward variable selection was performed to evaluate the association between gasping and outcomes. The candidate variables for multivariate logistic regression analysis using stepwise forward variable selection were age, sex, incidence of witnessed cardiac arrest, bystander CPR attempts, timing of cardiac arrest, epinephrine administration before arrival, ROSC during transportation, time from cardiac arrest to admission, cardiac rhythm at admission, epinephrine administration after arrival, gasping during resuscitation, therapeutic temperature management, intra-aortic balloon pumping, percutaneous coronary intervention, and time from admission to ECMO pump. All statistical analyses were conducted using SPSS Statistics version 25 (IBM Corp., Armonk, NY, USA) and EZR (Saitama Medical Centre, Jichi Medical University, Saitama, Japan), a graphical user interface for R (R Foundation for Statistical Computing, Vienna, Austria). All the tests were two-sided. A *p* value < 0.05 was considered statistically significant.

## Results

**Fig. 1:**
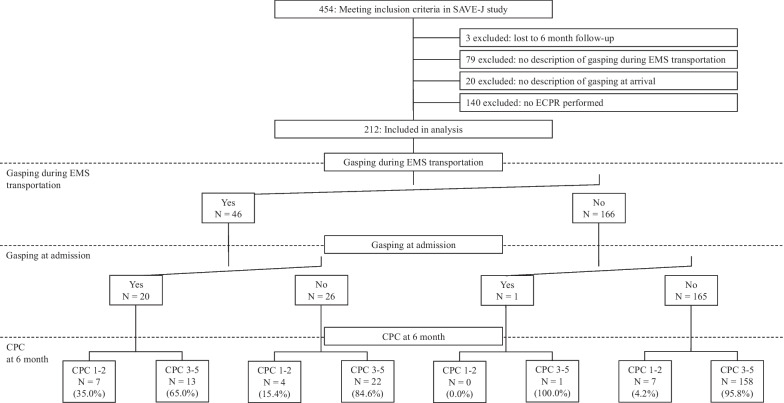
Patient enrollment, timing of gasping, and outcomes in ECPR group. *EMS* emergency medical service, *ECPR* extracorporeal cardiopulmonary resuscitation, *CPC* cerebral performance category

Of the 454 patients included in the SAVE-J study, 212 were enrolled. Three patients with unknown 6-month outcomes, 79 patients with unknown existence of gasping during emergency medical service (EMS) transport, 20 patients with unknown existence of gasping on hospital arrival, and 140 patients who did not undergo ECPR were excluded. Figure 1 shows the outcomes of each of the four groups according to the presence or absence of gasping during EMS transport and on arrival at the hospital. The acquisition rate of favorable neurological outcomes was 35% in patients with gasping during EMS transport and on arrival at the hospital.

A comparison of the characteristics of patients with or without gasping during resuscitation is shown in Table 1. Cases with gasping during resuscitation had more patients who developed cardiac arrest during EMS transport, had cardiac arrest rhythm as VF/pVT on hospital arrival, had favorable neurological outcomes, and had 6-month survival. A comparison of the neurological outcomes in patients undergoing ECPR is shown in Additional file 1. The presence of gasping at any time during resuscitation is associated with favorable neurological outcomes.

**Table 1:** Comparison between patients who underwent ECPR with and without gasping during resuscitation

	Presence of gasping during resuscitation	Absence of gasping during resuscitation	*p *value
	*n* = 47	*n* = 165	
Age (years), median [IQR]	58 [48, 65]	59 [48, 64]	0.678
Sex (female), *n* (%)	5 (10.6)	14 (8.5)	0.772
Witnessed cardiac arrest, *n* (%)	39 (83.0)	118 (71.5)	0.133
Bystander CPR attempt, *n* (%)			0.123
Yes	28 (59.6)	78 (47.3)	
No	18 (38.3)	86 (52.1)	
Unknown	1 (2.1)	1 (0.6)	
Occurrence of cardiac arrest during EMS activity, *n* (%)	4 (8.5)	3 (1.8)	0.045
Epinephrine administration before hospital arrival, *n* (%)			0.879
Yes	22 (46.8)	76 (46.1)	
No	24 (51.1)	81 (49.1)	
Unknown	1 (2.1)	8 (4.8)	
ROSC during EMS transportation, *n* (%)			0.643
Yes	11 (23.4)	29 (17.6)	
No	34 (72.3)	127 (77.0)	
Unknown	2 (4.3)	9 (5.5)	
Time from cardiac arrest to admission, median [IQR]	33 [23, 41]	31 [26, 38]	0.951
Cardiac rhythm at admission, *n* (%)			0.002
VF of pulseless VT	38 (80.9)	86 (52.1)	
PEA	4 (8.5)	38 (23.0)	
Asystole	5 (10.6)	41 (24.8)	
Epinephrine administration after hospital arrival, *n* (%)			0.542
Yes	40 (85.1)	128 (77.6)	
No	7 (14.9)	36 (21.8)	
Unknown	0 (0.0)	1 (0.6)	
Time from arrival to ECMO pump on, median [IQR]	22 [17, 37]	23 [17, 33]	0.869
Outcome at 6 months			
CPC 1–2, *n* (%)	11 (23.4)	7 (4.2)	< 0.001
Survival, *n* (%)	15 (31.9)	23 (13.9)	0.009

*IQR* interquartile range, *CPR* cardiopulmonary resuscitation, *ROSC* return of spontaneous circulation, *EMS* emergency medical service, *VF* ventricular fibrillation, *VT* ventricular tachycardia, *PEA* pulseless electrical activity, *ECMO* extracorporeal membrane oxygenation, *CPC* cerebral performance

### Association between neurological outcome and gasping in patients undergoing ECPR

Multivariate logistic regression analysis showed that gasping during resuscitation was independently associated with a favorable neurological outcome (odds ratio [OR], 10.58 [95% confidence interval (CI) 3.22–34.74]), after adjusting for age, ROSC during EMS transportation, and therapeutic temperature management (Table 2). Table 3 shows the unadjusted and adjusted ORs of favorable neurological outcomes with gasping during EMS transportation and/or on hospital arrival, with no gasping as the reference. The adjusted ORs for the presence of gasping either during EMS transport or on arrival at the hospital, or both, were 4.97 (95% CI 1.11–22.39) and 27.44 (95% CI 5.65–133.41), respectively.

**Table 2:** Logistic regression analysis of prognostic factors for favorable neurological outcomes in ECPR patients

Variables	Unadjusted OR (95% CI)	*p* value	Adjusted OR (95% CI)	*p* value
	*n* = 212		*n* = 207*	
Age (years)	0.95 (0.91–0.99)	0.006	0.93 (0.89–0.97)	0.002
Female sex	4.95 (1.54–15.87)	0.007		
witnessed cardiac arrest	1.25 (0.39–3.97)	0.707		
Bystander CPR attempt				
Yes	3.50 (1.10–11.10)	0.034		
No	1.00 (Ref.)	0.030		
Unknown	25.00 (1.31–475.97)	0.032		
Occurrence of cardiac arrest during EMS activity	1.84 (0.21–16.21)	0.582		
Epinephrine administration before hospital arrival				
Yes	1.59 (0.58–4.36)	0.367		
No	1.00 (Ref.)	0.642		
Unknown	1.75 (0.19–16.05)	0.621		
ROSC during transportation				
Yes	0.25 (0.03–1.95)	0.186	0.17 (0.02–1.74)	0.135
No	1.00 (Ref.)	0.240	1.00 (Ref.)	0.041
Unknown	2.16 (0.43–10.95)	0.351	8.13 (1.02–64.67)	0.048
Time from cardiac arrest to admission	0.96 (0.92–1.01)	0.104		
Cardiac rhythm at admission				
VF of pulseless VT	1.00 (Ref.)	0.372		
PEA	0.34 (0.07–1.53)	0.160		
Asystole	0.00 (0.00-)	0.997		
Epinephrine administration after hospital arrival				
Yes	1.31 (0.36–4.74)	0.683		
No	1.00 (Ref.)	0.920		
Unknown	0.00 (0.00-)	1.000		
Gasping during resuscitation	6.90 (2.50–19.02)	< 0.001	10.58 (3.22–34.74)	< 0.001
Therapeutic temperature management				
Yes	1.00 (Ref.)	1.000	1.00 (Ref.)	1.000
No	0.00 (0.00–)	0.997	0.00 (0.00–)	0.997
Unknown	0.00 (0.00–)	1.000	0.00 (0.00–)	1.000
Intra-aortic balloon pumping				
Yes	1.99 (0.44–9.02)	0.374		
No	1.00 (Ref.)	0.673		
Unknown	0.00 (0.00–)	0.999		
Percutaneous coronary intervention				
Yes	0.66 (0.24–1.80)	0.414		
No	1.00 (Ref.)	0.486		
Unknown	2.30 (0.23–22.63)	0.475		
Time from admission to ECMO pump on^a^	0.98 (0.95–1.03)	0.436		

Multivariate logistic analysis by replacing the variable "Therapeutic temperature management" with "Bystander CPR attempt" were also performed. The statistical significance of "Gasping during resuscitation" was similar. The result is shown in Additional file 10

*ECPR* extracorporeal cardiopulmonary resuscitation, *OR* odds ratio, *CI* confidence interval, *CPR* cardiopulmonary resuscitation, *EMS* emergency medical service, *ROSC* return of spontaneous circulation, *VF* ventricular fibrillation, *VT* ventricular tachycardia, *PEA* pulseless electrical activity, *ECMO* extracorporeal membrane oxygenation, *Ref.* reference

^a^Five data points were missing from admission to ECMO pump onset; thus, the multivariate analysis included 207 participants

**Table 3:** Odds ratios of neurological outcomes according to the recognition timing of gasping based on the absence of gasping in ECPR patients

	Unadjusted OR (95% CI)	*p* value	Adjusted OR (95%CI)	*p* value
	*n* = 212		*n* = 207^a^	
Without gasping during resuscitation	1.00 (Ref.)	< .001	1.00 (Ref.)	< .001
With gasping either during EMS transport or on hospital arrival	3.93 (1.07–14.46)	0.040	4.97 (1.11–22.39)	0.037
With gasping both during EMS transport and on hospital arrival	12.15 (3.70–39.97)	< .001	27.44 (5.65–133.41)	< .001

*ECPR* extracorporeal cardiopulmonary resuscitation, *EMS* emergency medical service, *OR* odds ratio, *CI* confidence interval, *Ref.* reference

^a^Five data points were missing from admission to ECMO pump onset; thus, the multivariate analysis included 207 participants

### Presence or absence of gasping and neurological outcome in all refractory VF/pVT cases

**Fig. 2:**
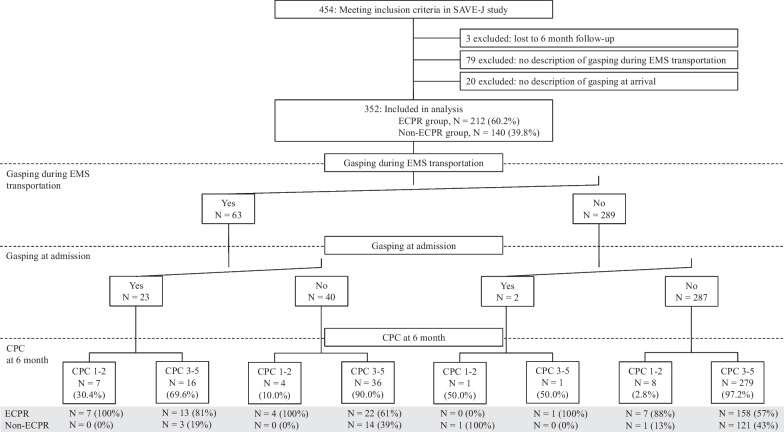
Patient enrollment, timing of gasping, and outcomes across all patients. *EMS* emergency medical service, *ECPR* extracorporeal cardiopulmonary resuscitation, *CPC* cerebral performance category

In total, 352 patients were included in the analysis after excluding three patients with unknown outcomes at 6 months, 79 patients with or without gasping during EMS transport, and 20 patients with or without gassing on arrival at the hospital (Fig. 2). A comparison of the characteristics of cases with or without gasping during resuscitation in all refractory VF/pVT cases is shown in Additional file 2, while the favorable and unfavorable neurological outcomes are shown in Additional file 3. Cases with gasping during resuscitation had more bystander CPR attempts, patients with cardiac arrest during EMS transport, cardiac arrest rhythm as VF/pVT on hospital arrival, favorable neurological outcomes, and 6-month survival. The presence of gasping at any time of resuscitation was associated with a favorable neurological outcome, similar to the case in the ECPR group.

Multivariate logistic regression analysis showed that gasping during resuscitation was independently associated with a favorable neurological outcome (OR 7.01 [95% CI 2.10–23.40]), after adjusting for age, female sex, cardiac arrest rhythm at admission, and therapeutic temperature management (Table 4). The odds of acquisition of favorable neurologic outcome with the existence of gasping both during EMS transport and on hospital arrival was 12.77 (95% CI 3.01–54.25) (Additional file 4).

**Table 4:** Logistic regression analysis of prognostic factors for favorable neurological outcomes in all patients

Variables	Unadjusted OR (95% CI)	*p* value	Adjusted OR (95% CI)	*p* value
	*n* = 352		*n* = 352	
Age (years)	0.94 (0.91–0.98)	0.001	0.94 (0.90–0.99)	0.009
Female sex	3.76 (1.35–10.42)	0.011	6.71 (1.42–31.74)	0.016
Witnessed cardiac arrest				
Yes	1.34 (0.44–4.12)	0.611		
No	1.00 (Ref.)	0.879		
Unknown	0.00 (0.00-)	1.000		
Bystander CPR attempt				
Yes	5.12 (1.66–15.76)	0.004		
No	1.00 (Ref.)	0.016		
Unknown	5.84 (0.58–58.45)	0.133		
Timing of cardiac arrest				
Before EMS arrival at scene	2.13 (0.25–)	0.488		
After EMS contact	1.00 (Ref.)	0.786		
Unknown	0.00 (0.00–)	1.000		
Epinephrine administration before hospital arrival				
Yes	1.68 (0.64–4.36)	0.290		
No	1.00 (Ref.)	0.360		
Unknown	2.83 (0.55–14.46)	0.212		
ROSC during transportation				
Yes	0.82 (0.23–2.91)	0.755		
No	1.00 (Ref.)	0.951		
Unknown	1.00 (0.22–4.60)	0.996		
Time from cardiac arrest to admission	0.96 (0.91–1.00)	0.048		
Cardiac rhythm at admission				
VF of pulseless VT	1.00 (Ref.)	0.157	1.00 (Ref.)	0.958
PEA	0.25 (0.06–1.09)	0.064	0.63 (0.12–3.26)	0.578
Asystole	0.00 (0.00–)	0.996	0.00 (0.00–)	0.996
Unknown	4.53 (0.39–52.60)	0.227	Inf^a^	0.995
Epinephrine administration after hospital arrival				
Yes	0.52 (0.17–1.65)	0.267		
No	1.00 (Ref.)	0.245		
Unknown	2.50 (0.22–28.13)	0.458		
Gasping during resuscitation	7.90 (3.08–20.25)	< 0.001	7.01 (2.10–23.40)	0.002
ECPR	6.40 (1.46–28.04)	0.014		
Therapeutic temperature management				
Yes	1.00 (Ref.)	1.000	1.00 (Ref.)	1.000
No	0.00 (0.00–)	0.995	0.00 (0.00–)	0.993
Unknown	0.00 (0.00–)	0.998	0.00 (0.00–)	0.997
Intra-aortic balloon pumping				
Yes	4.72 (1.35–)	0.015		
No	1.00 (Ref.)	0.052		
Unknown	0.00 (0.00–)	0.998		
Percutaneous coronary intervention				
Yes	1.23 (0.48–3.16)	0.665		
No	1.00 (Ref.)	0.348		
Unknown	0.26 (0.03–2.05)	0.201		

*ECPR* extracorporeal cardiopulmonary resuscitation, *OR* odds ratio, *CI* confidence interval, *CPR* cardiopulmonary resuscitation, *ROSC* return of spontaneous circulation, *VF* ventricular fibrillation, *VT* ventricular tachycardia, *PEA* pulseless electrical activity, *Ref* reference, *Inf* infinite

^a^The odds ratio was infinite, and the confidence interval could not be calculated

Of the 140 patients with refractory VF/pVT without ECPR, only two patients (1.4%) had favorable neurological outcomes (Additional file 5). Only one of the 18 patients with gasping during resuscitation had a favorable neurological outcome (Additional file 6). Additional files 7 and 8 show the comparison of neurological outcomes and logistic regression analysis results for the neurological outcomes of patients who did not undergo ECPR. Moreover, in comparing ECPR and non-ECPR in patients with gasping during resuscitation, 23.4% and 5.6% had favorable neurological outcomes in the ECPR and non-ECPR groups, respectively (*p* = 0.155, Additional file 9).

## Discussion

In patients with refractory VF/VT, the presence of gasping during resuscitation was an independent prognostic factor for better neurological outcomes at 6 months after onset. Patients who gasped both times during EMS activity and on hospital arrival, indicating continuous presentation of gasping, had a better neurological outcome than those with only one or the other.

In studies comparing ECPR and conventional CPR in patients with an initial VF/pVT rhythm, the outcomes of the ECPR group were generally favorable [8, 10, 19]. However, it is inconclusive whether ECPR should be performed in all refractory cardiac arrests with VF/pVT and if ROSC cannot be achieved before hospital arrival. The ARREST trial demonstrated the usefulness of ECPR in patients who could not obtain ROSC even after three defibrillation shocks, whose body morphology could accommodate ECMO, and whose estimated time to the emergency department was shorter than 30 min [10]. Although the neurological outcome at 6 months was promising (40%), the trial only included patients who required at least three defibrillation shocks and did not show the usefulness of ECPR on patients with refractory VF/pVT who did not obtain ROSC after 1–2 defibrillation shocks and were converted cardiac rhythm to PEA/asystole. The INCEPTION trial investigated whether ECPR could be compared with conventional CPR in patients with refractory VF/pVT who had witnessed and failed to obtain ROSC at 15 min ACLS [8]. As the neurological outcome for patients undergoing ECPR for the initial cardiac rhythm of VF/pVT and with witnessed onset was 20% (including approximately 26% of patients who did not receive ECPR), this could be lower in patients with early waveform VF/pVT without witnessed onset, even if ECPR is performed. The SAVE-J study targeted the initial cardiac rhythm of VF/pVT, cardiac arrest on arrival at the hospital, and no ROSC even after 15 min of ACLS after arrival [19]. Although the rate of obtaining a favorable neurological outcome for ECPR was 12.3% in the SAVE-J study, our results suggest a 23% chance of a favorable neurological outcome when gasping is observed during resuscitation, which may be a realistic strategy, including the implementation of ECPR. The initial cardiac rhythm of VF/pVT is considered an adequate adaptable indicator of ECPR, and we provide the additional finding that gasping during resuscitation increases the probability of acquiring favorable neurological outcomes.

To date, studies on the signs of life have focused on their presence or absence [11–14, 17]. However, in reality, these studies regarded cases with signs of life observed during ambulance transport as the same phenomenon, whether the sign of life was observed at the beginning and disappeared shortly during transport or it was observed continuously during transport. In the present study, gasping data were available during EMS transport and on arrival at the hospital. The OR for favorable neurological outcomes was 27.44 higher in patients with gasping at both times. Gasping at two different times may potentially suggest that gasping continuously exists during resuscitation. Continuous gasping expression suggests that CPR was consistently effective during resuscitation and generated sufficient cerebral blood flow to produce respiration. Thus, the present study demonstrates that continuous gasping expression is more relevant to favorable outcomes than gasping expression at a certain point. In addition, the relationship between the timing of emerging signs of life and outcomes should be examined in the future.

The SAVE-J study also provided data on cases, where ECPR was not performed for refractory VF/pVT [19]. Analysis of the usefulness of gasping during resuscitation in all refractory VF/pVT cases, including patients who did not undergo ECPR, showed that gasping during resuscitation was significantly associated with a favorable neurological outcome. Thus, gasping during resuscitation is a favorable factor in refractory VF/pVT, with or without ECPR. When ECPR was not introduced in patients with refractory VF/pVT with gasping, the acquisition rate of favorable neurological outcomes was 5.6%, compared with 23.4% in the ECPR implementation group (Additional file 9). Although ECPR appears to improve outcomes, more cases need to be included. In the future, the accumulation of outcomes in patients with refractory VF/pVT and gasping during resuscitation, with and without ECPR, will clarify whether gasping should be included in the criteria for ECPR implementation.

The present study had several limitations. First, the cases analyzed in the present study were from 2008 to 2011, making them somewhat older. Second, the number of patients with favorable neurological outcomes was relatively low at 18 (8.5%). This may have caused bias in the present analysis. Third, although a potential prognostic factor for OHCA is prehospital airway management [21], the information was not collected in the SAVE-J study [19] and, therefore, could not be included in the analysis. Fourth, in the multivariate analysis of neurologic outcomes (Table 2), "Therapeutic temperature management," which was not significantly different in the univariate analysis, was selected by the stepwise forward variable selection method. This was presumably included in the stepwise variable selection method because of its increased predictive (discriminative) power when combined with other variables. All patients in the favorable neurological outcome group received "Therapeutic temperature management," and those who did not receive "Therapeutic temperature management" were included only in the unfavorable neurological outcome group (Additional file 1). Thus, while it remains a presumption, the inclusion of "Therapeutic temperature management" as a variable in the multivariate logistic regression analysis may have been valuable in enhancing the predictive power and, consequently, selected for its contribution to the discrimination of outcomes. The forced entry of a variable (bystander CPR attempt), which was significantly different in the univariate analysis, instead of "Therapeutic temperature management," did not change the prognostic advantage of gasping (Additional file 10).

## Conclusion

Gasping during resuscitation is favorable in patients with refractory VF/pVT. Patients with refractory VF/pVT with continuously preserved gasping during EMS transportation to the hospital are expected to have more favorable outcomes.

### Supplementary Information

**Additional file 1: **Comparison of baseline characteristics according to neurological outcomes of ECPR patients.

**Additional file 2: **Comparison of patients with or without gasping during resuscitation.

**Additional file 3: **Comparison of baseline characteristics by neurological outcome in all patients.

**Additional file 4: **Odds ratios of neurological outcomes according to the recognition time of gasping based on the absence of gasping in all patients.

**Additional file 5: **Patient enrollment, timing, and outcomes in the non-ECPR group. EMS, emergency medical service; ECPR, extracorporeal cardiopulmonary resuscitation; CPC, cerebral performance category

**Additional file 6: **Comparison of patients with or without gasping during resuscitation who did not undergo ECPR

**Additional file 7: **Comparison of baseline characteristics by neurological outcomes in patients without ECPR.

**Additional file 8: **Logistic regression analysis of prognostic factors for favorable neurological outcomes in patients without ECPR.

**Additional file 9: **Comparison between ECPR and non-ECPR in patients who gasped during resuscitation.

**Additional file 10: **Logistic regression analysis of prognostic factors for favorable neurological outcomes in ECPR patients (Usage of "Bystander CPR attempt" instead of "Therapeutic temperature management" as variable).

## Data Availability

The data sets during and/or analyzed during the current study are available from the corresponding author on reasonable request.

## Abbreviations

ACLS: Advanced cardiac life support
CI: Confidence interval
CPC: Cerebral performance category
CPR: Cardiopulmonary resuscitation
ECPR: Extracorporeal CPR
EMS: Emergency medical service
OHCA: Out-of-hospital cardiac arrest
OR: Odd ratio
pVT: Pulseless ventricular tachycardia
ROSC: Restoration of spontaneous circulation
SAVE-J: Study on advanced cardiac life support for ventricular fibrillation with extracorporeal circulation in Japan
VF: Ventricular fibrillation
