# Supplementary material for: Long-term prognostic significance of gasping in out-of-hospital cardiac arrest patients undergoing extracorporeal cardiopulmonary resuscitation: a post hoc analysis of a multi-center prospective cohort study
Source: J Intensive Care. 2023 Oct 6;11:43. doi: 10.1186/s40560-023-00692-1 (PMC10559458; doi:10.1186/s40560-023-00692-1)
Supplement: Supplementary file 1 — Additional file 1: Comparison of baseline characteristics according to neurological outcomes of ECPR patients. [file 40560_2023_692_MOESM1_ESM.docx]

**Additional File 1.** Comparison of baseline characteristics according to neurological outcomes of ECPR patients

|  | **Favorable** | **Unfavorable** | ***p*** |
| --- | --- | --- | --- |
|  | **outcome** | **outcome** | **value** |
|  | **n = 18** | **n = 194** |  |
| Age (years), median [IQR] | 50 [34, 62] | 59 [49, 65] | 0.026 |
| Sex (female), *n* (%) | 5 (27.8) | 14 (7.2) | 0.014 |
| Witnessed cardiac arrest, *n* (%) | 14 (77.8) | 143 (73.7) | 1.000 |
| Bystander CPR attempt, *n* (%) |  |  | 0.018 |
| Yes | 13 (72.2) | 93 (47.9) |  |
| No | 4 (22.2) | 100 (51.5) |  |
| Unknown | 1 (5.6) | 1 (0.5) |  |
| Occurrence of cardiac arrest during EMS activity, *n* (%) | 1 (5.6) | 6 (3.1) | 0.468 |
| Epinephrine administration before hospital arrival, *n* (%) |  |  | 0.489 |
| Yes | 10 (55.6) | 88 (45.4) |  |
| No | 7 (38.9) | 98 (50.5) |  |
| Unknown | 1 (5.6) | 8 (4.1) |  |
| ROSC during EMS transportation, *n* (%) |  |  | 0.133 |
| Yes | 1 (5.6) | 39 (20.1) |  |
| No | 15 (83.3) | 146 (75.3) |  |
| Unknown | 2 (11.1) | 9 (4.6) |  |
| Time from cardiac arrest to arrival (min.), median [IQR] | 26 [19, 42] | 32 [26, 39] | 0.093 |
| Cardiac rhythm at admission, *n* (%) |  |  | <.001 |
| VF of pulseless VT | 16 (88.9) | 108 (55.7) |  |
| PEA | 2 (11.1) | 40 (20.6) |  |
| Asystole | 0 (0.0) | 46 (23.7) |  |
| Unknown |  |  |  |
| Epinephrine administration after hospital arrival, *n* (%) |  |  | 1.000 |
| Yes | 15 (83.3) | 153 (78.9) |  |
| No | 3 (16.7) | 40 (20.6) |  |
| Unknown | 0 (0.0) | 1 (0.5) |  |
| Gasping during resuscitation, *n* (%) |  |  |  |
| Gasping during EMS transportation | 11 (61.1) | 35 (18.0) | <.001 |
| Gasping at arrival | 7 (38.9) | 14 (7.2) | <.001 |
| Gasping during EMS transportation or at arrival | 11 (61.1) | 36 (18.6) | <.001 |
| Gasping both during EMS transportation and at arrival | 7 (38.9) | 13 (6.7) | <.001 |
| Time from arrival to ECMO pump on (min.), median [IQR]* | 23 [16, 32] | 23 [17, 33] | 0.532 |
| Therapeutic temperature management, *n* (%) |  |  | 0.019 |
| Yes | 18 (100.0) | 142 (73.2) |  |
| No | 0 (0.0) | 51 (20.3) |  |
| Unknown | 0 (0.0) | 1 (0.5) |  |
| Percutaneous coronary intervention, *n* (%) |  |  | 0.288 |
| Yes | 7 (38.9) | 98 (50.5) |  |
| No | 10 (55.6) | 92 (47.4) |  |
| Unknown | 1 (5.6) | 4 (2.1) |  |
| Intra-aortic balloon pumping, *n* (%) |  |  | 0.644 |
| Yes | 16 (88.9) | 153 (78.9) |  |
| No | 2 (11.1) | 38 (19.6) |  |
| Unknown | 0 (0.0) | 3 (1.5) |  |

*Five data points were missing from admission to ECMO pump onset.

IQR, interquartile range; CPR, cardiopulmonary resuscitation; ROSC, return of spontaneous circulation; EMS, emergency medical service; VF, ventricular fibrillation; VT, ventricular tachycardia; PEA, pulseless electrical activity; ECMO, extracorporeal membrane oxygenation; CPC, cerebral performance.
