# Supplementary material for: Long-term prognostic significance of gasping in out-of-hospital cardiac arrest patients undergoing extracorporeal cardiopulmonary resuscitation: a post hoc analysis of a multi-center prospective cohort study
Source: J Intensive Care. 2023 Oct 6;11:43. doi: 10.1186/s40560-023-00692-1 (PMC10559458; doi:10.1186/s40560-023-00692-1)
Supplement: Supplementary file 2 — Additional file 2: Comparison of patients with or without gasping during resuscitation. [file 40560_2023_692_MOESM2_ESM.docx]

**Additional File 2.** Comparison of patients with or without gasping during resuscitation

|  | **presence of gasping** | **absence of gasping** | **p** |
| --- | --- | --- | --- |
|  | **during resuscitation** | **during resuscitation** | **value** |
|  | **n = 65** | **n = 287** |  |
| Age (years), median [IQR] | 60 [50, 65] | 60 [50, 66] | 0.546 |
| Sex (female), *n* (%) | 7 (10.8) | 33 (11.5) | 1.000 |
| Witnessed cardiac arrest, *n* (%) |  |  | 0.354 |
| Yes | 53 (81.5) | 211 (73.5) |  |
| No | 12 (18.5) | 75 (26.1) |  |
| Unknown | 0 (0.0) | 1 (0.3) |  |
| Bystander CPR attempt, *n* (%) |  |  | 0.009 |
| Yes | 35 (53.8) | 117 (40.8) |  |
| No | 26 (40.0) | 165 (57.5) |  |
| Unknown | 4 (6.2) | 5 (1.7) |  |
| Timing of cardiac arrest, *n* (%) |  |  | 0.022 |
| before EMS arrival at scene | 60 (92.3) | 282 (98.3) |  |
| after EMS contact | 5 (7.7) | 4 (1.4) |  |
| Unknown | 0 (0.0) | 1 (0.3) |  |
| Epinephrine administration before hospital arrival, *n* (%) |  |  | 0.919 |
| Yes | 25 (38.5) | 120 (41.8) |  |
| No | 37 (56.9) | 152 (53.0) |  |
| Unknown | 3 (4.6) | 15 (5.2) |  |
| ROSC during EMS transportation, *n* (%) |  |  | 0.192 |
| Yes | 16 (24.6) | 46 (16.0) |  |
| No | 45 (69.2) | 211 (73.5) |  |
| Unknown | 4 (6.2) | 30 (10.5) |  |
| Time from cardiac arrest to admission (min.), median [IQR] | 32 [24, 40] | 32 [26, 40] | 0.492 |
| Cardiac rhythm at admission, *n* (%) |  |  | <.001 |
| VF of pulseless VT | 46 (70.8) | 125 (43.6) |  |
| PEA | 8 (12.3) | 68 (23.7) |  |
| Asystole | 10 (15.4) | 92 (32.1) |  |
| Unknown | 1 (1.5) | 2 (0.7) |  |
| Epinephrine administration after hospital arrival, *n* (%) |  |  | 0.932 |
| Yes | 57 (87.7) | 246 (85.7) |  |
| No | 7 (10.8) | 37 (12.9) |  |
| Unknown | 1 (1.5) | 4 (1.4) |  |
| ECPR, *n* (%) | 47 (72.3) | 165 (57.5) | 0.035 |
| Outcome at 6 months |  |  |  |
| CPC 1-2, *n* (%) | 12 (18.5) | 8 (2.8) | <.001 |
| Survival, *n* (%) | 17 (26.2) | 25 (8.7) | <.001 |

IQR, interquartile range; CPR, cardiopulmonary resuscitation; EMS, emergency medical service; ROSC, return of spontaneous circulation min; VF, ventricular fibrillation; VT, ventricular tachycardia; PEA, pulseless electrical activity; ECPR, extracorporeal cardiopulmonary resuscitation; CPC, cerebral performance category.
