# Supplementary material for: Long-term prognostic significance of gasping in out-of-hospital cardiac arrest patients undergoing extracorporeal cardiopulmonary resuscitation: a post hoc analysis of a multi-center prospective cohort study
Source: J Intensive Care. 2023 Oct 6;11:43. doi: 10.1186/s40560-023-00692-1 (PMC10559458; doi:10.1186/s40560-023-00692-1)
Supplement: Supplementary file 3 — Additional file 3: Comparison of baseline characteristics by neurological outcome in all patients. [file 40560_2023_692_MOESM3_ESM.docx]

**Additional File 3.** Comparison of baseline characteristics by neurological outcome in all patients

|  | **Favorable** | **Unfavorable** | ***p*** |
| --- | --- | --- | --- |
|  | **outcome** | **outcome** | **value** |
|  | **n = 20** | **n = 332** |  |
| Age (years), median [IQR] | 50 [34, 61] | 60 [51, 66] | 0.004 |
| Sex (female), *n* (%) | 6 (30.0) | 34 (10.2) | 0.017 |
| Witnessed cardiac arrest, *n* (%) |  |  | 0.804 |
| Yes | 16 (80.0) | 248 (74.7) |  |
| No | 4 (20.0) | 83 (25.0) |  |
| Unknown | 0 (0.0) | 1 (0.3) |  |
| Bystander CPR attempt, *n* (%) |  |  | 0.005 |
| Yes | 15 (75.0) | 137 (41.3) |  |
| No | 4 (20.0) | 187 (56.3) |  |
| Unknown | 1 (5.0) | 8 (2.4) |  |
| Timing of cardiac arrest, *n* (%) |  |  | 0.447 |
| before EMS arrival at scene | 19 (95.0) | 323 (97.3) |  |
| after EMS contact | 1 (5.0) | 8 (2.4) |  |
| unknown | 0 (0.0) | 1 (0.3) |  |
| Epinephrine administration before hospital arrival, *n* (%) |  |  | 0.232 |
| Yes | 10 (50.0) | 135 (40.7) |  |
| No | 8 (40.0) | 181 (54.5) |  |
| Unknown | 2 (10.0) | 16 (4.8) |  |
| ROSC during EMS transportation, *n* (%) |  |  | 1.000 |
| Yes | 3 (15.0) | 59 (17.8) |  |
| No | 15 (75.0) | 241 (72.6) |  |
| Unknown | 2 (10.0) | 32 (9.6) |  |
| Time from cardiac arrest to arrival (min.), median [IQR] | 28 [19, 40] | 33 [26, 40] | 0.059 |
| Cardiac rhythm at admission, *n* (%) |  |  | <.001 |
| VF of pulseless VT | 17 (85.0) | 154 (46.4) |  |
| PEA | 2 (10.0) | 74 (22.3) |  |
| Asystole | 0 (0.0) | 102 (30.7) |  |
| Unknown | 1 (5.0) | 2 (0.6) |  |
| Epinephrine administration after hospital arrival |  |  | 0.113 |
| Yes | 15 (75.0) | 288 (86.7) |  |
| No | 4 (20.0) | 40 (12.0) |  |
| Unknown | 1 (5.0) | 4 (1.2) |  |
| Gasping during resuscitation, *n* (%) |  |  |  |
| Gasping during EMS transportation | 11 (55.0) | 52 (15.7) | <.001 |
| Gasping at arrival | 8 (40.0) | 17 (5.1) | <.001 |
| Gasping during EMS transportation or at arrival | 12 (60.0) | 53 (16.0) | <.001 |
| Gasping both during EMS transportation and at arrival | 7 (35.0) | 16 (4.8) | <.001 |
| ECPR, *n* (%) | 18 (90.0) | 194 (58.4) | 0.004 |
| Therapeutic temperature management, *n* (%) |  |  | <.001 |
| Yes | 20 (100.0) | 145 (43.7) |  |
| No | 0 (0.0) | 149 (44.9) |  |
| Unknown | 0 (0.0) | 38 (11.4) |  |
| Percutaneous coronary intervention, *n* (%) |  |  | 0.333 |
| Yes | 8 (40.0) | 101 (30.4) |  |
| No | 11 (55.0) | 171 (51.5) |  |
| Unknown | 1 (5.0) | 60 (18.1) |  |
| Intra-aortic balloon pumping, *n* (%) |  |  | 0.004 |
| Yes | 17 (85.0) | 156 (47.0) |  |
| No | 3 (15.0) | 130 (39.2) |  |
| Unknown | 0 (0.0) | 46 (13.9) |  |

IQR: interquartile range, CPR: cardiopulmonary resuscitation, ROSC: return of spontaneous circulation, EMS: emergency medical service, VF: ventricular fibrillation, VT: ventricular tachycardia, PEA: pulseless electrical activity, ECPR: extracorporeal cardiopulmonary resuscitation, CPC: cerebral performance category
