# Supplementary material for: Long-term prognostic significance of gasping in out-of-hospital cardiac arrest patients undergoing extracorporeal cardiopulmonary resuscitation: a post hoc analysis of a multi-center prospective cohort study
Source: J Intensive Care. 2023 Oct 6;11:43. doi: 10.1186/s40560-023-00692-1 (PMC10559458; doi:10.1186/s40560-023-00692-1)
Supplement: Supplementary file 4 — Additional file 4: Odds ratios of neurological outcomes according to the recognition time of gasping based on the absence of gasping in all patients. [file 40560_2023_692_MOESM4_ESM.docx]

**Additional File 4.** Odds ratios of neurological outcomes according to the recognition time of gasping based on the absence of gasping in all patients

|  | **Unadjusted OR (95% CI)** | ***p*** | **Adjusted OR (95%CI)** | ***p*** |
| --- | --- | --- | --- | --- |
|  | **n = 352** | **value** | **n = 352** | **value** |
| Without gasping during resuscitation | Ref. | <.001 | Ref. | 0.002 |
| With gasping either during EMS transport or at the arrival | 4.71 (1.46-15.17) | 0.009 | 3.56 (0.73-17.39) | 0.116 |
| With gasping both during EMS transport and at the arrival | 15.26 (4.92-47.36) | <.001 | 12.77 (3.01-54.25) | 0.001 |

ECPR, extracorporeal cardiopulmonary resuscitation; EMS, emergency medical service; OR, odds ratio; CI, confidence interval. Ref, reference.
