# Supplementary material for: Long-term prognostic significance of gasping in out-of-hospital cardiac arrest patients undergoing extracorporeal cardiopulmonary resuscitation: a post hoc analysis of a multi-center prospective cohort study
Source: J Intensive Care. 2023 Oct 6;11:43. doi: 10.1186/s40560-023-00692-1 (PMC10559458; doi:10.1186/s40560-023-00692-1)
Supplement: Supplementary file 5 — Additional file 5: Patient enrollment, timing, and outcomes in the non-ECPR group. EMS, emergency medical service; ECPR, extracorporeal cardiopulmonary resuscitation; CPC, cerebral performance category [file 40560_2023_692_MOESM5_ESM.docx]

**Additional File 5**. Patient enrollment, timing, and outcomes in the non-ECPR group


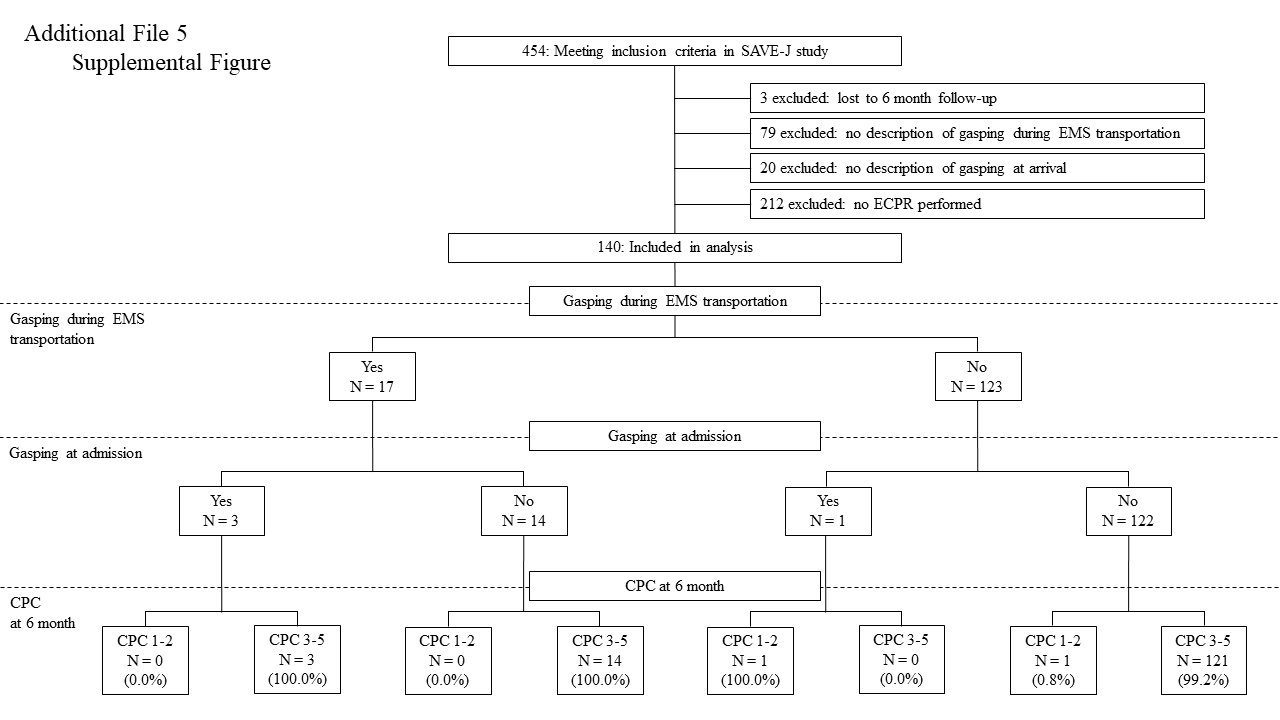


EMS, emergency medical service; ECPR, extracorporeal cardiopulmonary resuscitation; CPC, cerebral performance category
