# Supplementary material for: Long-term prognostic significance of gasping in out-of-hospital cardiac arrest patients undergoing extracorporeal cardiopulmonary resuscitation: a post hoc analysis of a multi-center prospective cohort study
Source: J Intensive Care. 2023 Oct 6;11:43. doi: 10.1186/s40560-023-00692-1 (PMC10559458; doi:10.1186/s40560-023-00692-1)
Supplement: Supplementary file 6 — Additional file 6: Comparison of patients with or without gasping during resuscitation who did not undergo ECPR [file 40560_2023_692_MOESM6_ESM.docx]

**Additional File 6.** Comparison of patients with or without gasping during resuscitation who did not undergo ECPR

|  | **presence of gasping** | **absence of gasping** | ***p*** |
| --- | --- | --- | --- |
|  | **during resuscitation** | **during resuscitation** | **value** |
|  | **n = 18** | **n = 122** |  |
| Age (years), median [IQR] | 62 [58, 66] | 62 [53, 69] | 0.179 |
| Sex (female), *n* (%) | 2 (11.1) | 19 (15.6) | 1.000 |
| Witnessed cardiac arrest, *n* (%) |  |  | 1.000 |
| Yes | 14 (77.8) | 93 (76.2) |  |
| No | 4 (22.2) | 28 (23.0) |  |
| Unknown | 0 (0.0) | 1 (0.8) |  |
| Bystander CPR attempt, *n* (%) |  |  | 0.036 |
| Yes | 7 (38.9) | 39 (32.0) |  |
| No | 8 (44.4) | 79 (64.8) |  |
| Unknown | 3 (16.7) | 4 (3.3) |  |
| Timing of cardiac arrest, *n* (%) |  |  | 0.340 |
| Before EMS arrival | 17 (94.4) | 120 (98.4) |  |
| During EMS transport | 1 (5.6) | 1 (0.8) |  |
| Unknown | 1 (5.6) | 1 (0.8) |  |
| Epinephrine administration before hospital arrival, *n* (%) | | | 0.184 |
| Yes | 3 (16.7) | 44 (36.1) |  |
| No | 13 (72.2) | 71 (58.2) |  |
| Unknown | 2 (11.1) | 7 (5.7) |  |
| ROSC during EMS transportation, *n* (%) | |  | 0.274 |
| Yes | 5 (27.8) | 17 (13.9) |  |
| No | 11 (61.1) | 84 (68.9) |  |
| Unknown | 2 (11.1) | 21 (17.2) |  |
| Time from cardiac arrest to admission, median [IQR] | 33 [23, 41] | 34 [27, 41] | 0.923 |
| Cardiac rhythm at admission, *n* (%) |  |  | 0.341 |
| VF of pulseless VT | 8 (44.4) | 39 (32.0) |  |
| PEA | 4 (22.2) | 30 (24.6) |  |
| Asystole | 5 (27.8) | 51 (41.8) |  |
| Unknown | 1 (5.6) | 2 (1.6) |  |
| Epinephrine administration after hospital arrival, *n* (%) | | | 0.503 |
| Yes | 17 (94.4) | 118 (96.7) |  |
| No | 0 (0.0) | 1 (0.8) |  |
| Unknown | 1 (5.6) | 3 (2.5) |  |
| Outcome at 6 months |  |  |  |
| CPC 1-2, *n* (%) | 1 (5.6) | 1 (0.8) | 0.241 |
| Survival, *n* (%) | 2 (11.1) | 2 (1.6) | 0.080 |

IQR, interquartile range; CPR, cardiopulmonary resuscitation; ROSC, return of spontaneous circulation; EMS, emergency medical service; VF, ventricular fibrillation; VT, ventricular tachycardia; PEA, pulseless electrical activity; CPC, cerebral performance category.
