# Supplementary material for: Long-term prognostic significance of gasping in out-of-hospital cardiac arrest patients undergoing extracorporeal cardiopulmonary resuscitation: a post hoc analysis of a multi-center prospective cohort study
Source: J Intensive Care. 2023 Oct 6;11:43. doi: 10.1186/s40560-023-00692-1 (PMC10559458; doi:10.1186/s40560-023-00692-1)
Supplement: Supplementary file 7 — Additional file 7: Comparison of baseline characteristics by neurological outcomes in patients without ECPR. [file 40560_2023_692_MOESM7_ESM.docx]

**Additional File 7.** Comparison of baseline characteristics by neurological outcomes in patients without ECPR.

|  | **Favorable** | **Unfavorable** | ***p*** |
| --- | --- | --- | --- |
|  | **outcome** | **outcome** | **value** |
|  | **n = 2** | **n = 138** |  |
| Age (years), median [IQR] | 49 [38, -] | 62 [54, 68] | 0.179 |
| Sex (female), *n* (%) | 1 (50.0) | 20 (14.5) | 0.014 |
| Witnessed cardiac arrest, *n* (%) |  |  | 1.000 |
| Yes | 2 (100.0) | 105 (76.1) |  |
| No | 0 (0.0) | 32 (23.2) |  |
| Unknown | 0 (0.0) | 1 (0.7) |  |
| Bystander CPR attempt, *n* (%) |  |  | 0.204 |
| Yes | 2 (100.0) | 44 (31.9) |  |
| No | 0 (0.0) | 87 (63.0) |  |
| Unknown | 0 (0.0) | 7 (5.1) |  |
| Timing of cardiac arrest, *n* (%) |  |  | 1.000 |
| Before EMS arrival | 2 (100.0) | 135 (97.8) |  |
| During EMS transport | 0 (0.0) | 2 (1.4) |  |
| Unknown | 0 (0.0) | 1 (0.7) |  |
| Epinephrine administration before hospital arrival, *n* (%) |  |  | 0.125 |
| Yes | 0 (0.0) | 47 (34.1) |  |
| No | 1 (50.0) | 83 (60.1) |  |
| Unknown | 1 (50.0) | 8 (5.8) |  |
| ROSC during EMS transportation, *n* (%) |  |  | 0.024 |
| Yes | 2 (100.0) | 20 (14.5) |  |
| No | 0 (0.0) | 95 (68.8) |  |
| Unknown | 0 (0.0) | 23 (16.7) |  |
| Time from cardiac arrest to arrival (min.), median [IQR] | 33 [30, -] | 34 [27, 41] | 0.923 |
| Cardiac rhythm at admission, *n* (%) |  |  | 0.025 |
| VF of pulseless VT | 1 (50.0) | 46 (33.3) |  |
| PEA | 0 (0.0) | 34 (24.6) |  |
| Asystole | 0 (0.0) | 56 (40.6) |  |
| Unknown | 1 (50.0) | 2 (1.4) |  |
| Epinephrine administration after hospital arrival, n (%) |  |  | <.001 |
| Yes | 0 (0.0) | 135 (97.8) |  |
| No | 1 (50.0) | 0 (0.0) |  |
| Unknown | 1 (50.0) | 3 (2.2) |  |
| Gasping during resuscitation, n (%) |  |  |  |
| Gasping during EMS transportation | 0 (0.0) | 17 (12.3) | 1.000 |
| Gasping at arrival | 1 (50.0) | 3 (2.2) | 0.057 |
| Gasping during EMS transportation or at arrival | 1 (50.0) | 17 (12.3) | 0.241 |
| Gasping both during EMS transportation and at arrival | 0 (0.0) | 3 (2.2) | 1.000 |
| Therapeutic temperature management, *n* (%) |  |  | 0.001 |
| Yes | 2 (100.0) | 3 (2.2) |  |
| No | 0 (0.0) | 98 (71.0) |  |
| Unknown | 0 (0.0) | 37 (26.8) |  |
| Percutaneous coronary intervention, *n* (%) |  |  | 0.057 |
| Yes | 1 (50.0) | 3 (2.2) |  |
| No | 1 (50.0) | 79 (57.2) |  |
| Unknown | 0 (0.0) | 56 (40.6) |  |
| Intra-aortic balloon pumping, *n* (%) |  |  | 0.057 |
| Yes | 1 (50.0) | 3 (2.2) |  |
| No | 1 (50.0) | 92 (66.7) |  |
| Unknown | 0 (0.0) | 43 (31.2) |  |

IQR, interquartile range; CPR, cardiopulmonary resuscitation; ROSC, return of spontaneous circulation; EMS, emergency medical service; VF, ventricular fibrillation; VT, ventricular tachycardia; PEA, pulseless electrical activity; CPC, cerebral performance category.
