# Supplementary material for: Long-term prognostic significance of gasping in out-of-hospital cardiac arrest patients undergoing extracorporeal cardiopulmonary resuscitation: a post hoc analysis of a multi-center prospective cohort study
Source: J Intensive Care. 2023 Oct 6;11:43. doi: 10.1186/s40560-023-00692-1 (PMC10559458; doi:10.1186/s40560-023-00692-1)
Supplement: Supplementary file 8 — Additional file 8: Logistic regression analysis of prognostic factors for favorable neurological outcomes in patients without ECPR. [file 40560_2023_692_MOESM8_ESM.docx]

**Additional File 8.** Logistic regression analysis of prognostic factors for favorable neurological outcomes in patients without ECPR

|  | **Unadjusted OR** | ***p*** | **Adjusted OR** | ***p*** |
| --- | --- | --- | --- | --- |
| **variables** | **(95% CI)** | **value** | **(95% CI)** | **value** |
|  | **n = 140** |  | **n = 140** |  |
| Age (years) | 0.94 (0.85-1.03) | 0.198 |  |  |
| Female sex | 5.90 (0.35-98.20) | 0.216 |  |  |
| witnessed cardiac arrest |  |  |  |  |
| Yes | Inf* | 0.998 |  |  |
| No | 1.00 (Ref.) | 1.000 |  |  |
| Unknown | 1.00 (0.00- ) | 1.000 |  |  |
| Bystander CPR attempt |  |  |  |  |
| Yes | Inf* | 0.997 |  |  |
| No | 1.00 (Ref.) | 1.000 |  |  |
| Unknown | 1.00 (0.00- ) | 1.000 |  |  |
| Timing of cardiac arrest |  |  |  |  |
| Before EMS arrival | 1.00 (Ref.) | 1.000 |  |  |
| During EMS transport | 0.00 (0.00- ) | 1.000 |  |  |
| Unknown | 0.00 (0.00- ) | 1.000 |  |  |
| Epinephrine administration before hospital arrival | | |  |  |
| Yes | 0.00 (0.00- ) | 0.998 |  |  |
| No | 1.00 (Ref.) | 0.278 |  |  |
| Unknown | 10.34 (0.59-182.11) | 0.110 |  |  |
| ROSC during transportation |  |  |  |  |
| Yes | Inf* | 0.996 |  |  |
| No | 1.00 (Ref.) | 1.000 |  |  |
| Unknown | 1.00 (0.00- ) | 1.000 |  |  |
| Time from cardiac arrest to admission | 0.99 (0.84-1.16) | 0.902 |  |  |
| Cardiac rhythm at admission |  |  |  |  |
| VF of pulseless VT | 1.00 (Ref.) | 0.273 | 1.00 (Ref.) | 1.000 |
| PEA | 0.00 (0.00- ) | 0.998 | 1.50 (0.00- ) | 1.000 |
| Asystole | 0.00 (0.00- ) | 0.997 | 0.00 (0.00- ) | 1.000 |
| Unknown | 23.00 (1.02-516.93) | 0.048 | 2112.49 (0.00- ) | 1.000 |
| Epinephrine administration after hospital arrival | |  |  |  |
| Yes | 0.00 (0.00- ) | 0.999 | 0.00 (0.00- ) | 0.999 |
| No | 1.00 (Ref.) | 1.000 | 1.00 (Ref.) | 1.000 |
| Unknown | 0.00 (0.00- ) | 1.000 | 0.00 (0.00- ) | 1.000 |
| Gasping during resuscitation | 7.12 (0.43-119.16) | 0.172 | 363.08 (0.00- ) | 1.000 |
| Therapeutic temperature management | |  |  |  |
| Yes | 1.00 (ref.) | 1.000 |  |  |
| No | 0.00 (0.00- ) | 0.996 |  |  |
| Unknown | 0.00 (0.00- ) | 0.997 |  |  |
| Intra-aortic balloon pumping |  |  |  |  |
| Yes | 30.67 (1.53-616.49) | 0.025 |  |  |
| No | 1.00 (Ref.) | 0.082 |  |  |
| Unknown | 0.00 (0.00- ) | 0.998 |  |  |
| Percutaneous coronary intervention | |  |  |  |
| Yes | 26.33 (1.31-529.98) | 0.033 |  |  |
| No | 1.00 (Ref.) | 0.102 |  |  |
| Unknown | 0.00 (0.00- ) | 0.997 |  |  |

*The odds ratio was infinite, and the confidence interval could not be calculated.

ECPR, extracorporeal cardiopulmonary resuscitation; OR, odds ratio; CI, confidence interval; CPR, cardiopulmonary resuscitation; ROSC, return of spontaneous circulation; VF, ventricular fibrillation; VT, ventricular tachycardia; PEA, pulseless electrical activity; Ref., reference; Inf, infinite.
