# Supplementary material for: Long-term prognostic significance of gasping in out-of-hospital cardiac arrest patients undergoing extracorporeal cardiopulmonary resuscitation: a post hoc analysis of a multi-center prospective cohort study
Source: J Intensive Care. 2023 Oct 6;11:43. doi: 10.1186/s40560-023-00692-1 (PMC10559458; doi:10.1186/s40560-023-00692-1)
Supplement: Supplementary file 9 — Additional file 9: Comparison between ECPR and non-ECPR in patients who gasped during resuscitation. [file 40560_2023_692_MOESM9_ESM.docx]

**Additional File 9.** Comparison between ECPR and non-ECPR in patients who gasped during resuscitation.

|  | **ECPR group** | **non-ECPR group** | **p** |
| --- | --- | --- | --- |
|  | **n = 47** | **n = 18** | **value** |
| Outcome at 6 months |  |  |  |
| CPC 1-2, *n* (%) | 11 (23.4) | 1 (5.6) | 0.155 |
| Survival, *n* (%) | 15 (31.9) | 2 (11.1) | 0.119 |

ECPR, extracorporeal cardiopulmonary resuscitation; CPC, Cerebral Performance Category.
