# Supplementary material for: Long-term prognostic significance of gasping in out-of-hospital cardiac arrest patients undergoing extracorporeal cardiopulmonary resuscitation: a post hoc analysis of a multi-center prospective cohort study
Source: J Intensive Care. 2023 Oct 6;11:43. doi: 10.1186/s40560-023-00692-1 (PMC10559458; doi:10.1186/s40560-023-00692-1)
Supplement: Supplementary file 10 — Additional file 10: Logistic regression analysis of prognostic factors for favorable neurological outcomes in ECPR patients (Usage of "Bystander CPR attempt" instead of "Therapeutic temperature management" as variable). [file 40560_2023_692_MOESM10_ESM.docx]

**Additional File 10 (Table 2B).** Logistic regression analysis of prognostic factors for favorable neurological outcomes in ECPR patients

|  | **Unadjusted OR** | ***p*** | **Adjusted OR** | ***p*** |
| --- | --- | --- | --- | --- |
| **variables** | **(95% CI)** | **value** | **(95% CI)** | **value** |
|  | **n = 212** |  | **n = 207*** |  |
| Age (years) | 0.95 (0.91-0.99) | 0.006 | 0.94 (0.90-0.98) | 0.006 |
| Female sex | 4.95 (1.54-15.87) | 0.007 |  |  |
| witnessed cardiac arrest | 1.25 (0.39-3.97) | 0.707 |  |  |
| Bystander CPR attempt |  |  |  |  |
| Yes | 3.50 (1.10-11.10) | 0.034 | 3.17 (0.91-11.09) | 0.071 |
| No | 1.00 (Ref.) | 0.030 | 1.00 (Ref.) | 0.103 |
| Unknown | 25.00 (1.31-475.97) | 0.032 | 29.62 (0.36-2421.38) | 0.132 |
| Occurrence of cardiac arrest during EMS activity | 1.84 (0.21-16.21) | 0.582 |  |  |
| Epinephrine administration before hospital arrival | | |  |  |
| Yes | 1.59 (0.58-4.36) | 0.367 |  |  |
| No | 1.00 (Ref.) | 0.642 |  |  |
| Unknown | 1.75 (0.19-16.05) | 0.621 |  |  |
| ROSC during transportation |  |  |  |  |
| Yes | 0.25 (0.03-1.95) | 0.186 | 0.13 (0.01-1.25) | 0.077 |
| No | 1.00 (Ref.) | 0.240 | 1.00 (Ref.) | 0.033 |
| Unknown | 2.16 (0.43-10.95) | 0.351 | 5.52 (0.93-32.92) | 0.061 |
| Time from cardiac arrest to admission | 0.96 (0.92-1.01) | 0.104 |  |  |
| Cardiac rhythm at admission |  |  |  |  |
| VF of pulseless VT | 1.00 (Ref.) | 0.372 |  |  |
| PEA | 0.34 (0.07-1.53) | 0.160 |  |  |
| Asystole | 0.00 (0.00- ) | 0.997 |  |  |
| Epinephrine administration after hospital arrival | |  |  |  |
| Yes | 1.31 (0.36-4.74) | 0.683 |  |  |
| No | 1.00 (Ref.) | 0.920 |  |  |
| Unknown | 0.00 (0.00- ) | 1.000 |  |  |
| Gasping during resuscitation | 6.90 (2.50-19.02) | <.001 | 8.04 (2.56-25.20) | <.001 |
| Therapeutic temperature management | |  |  |  |
| Yes | 1.00 (Ref.) | 1.000 |  |  |
| No | 0.00 (0.00- ) | 0.997 |  |  |
| Unknown | 0.00 (0.00- ) | 1.000 |  |  |
| Intra-aortic balloon pumping |  |  |  |  |
| Yes | 1.99 (0.44-9.02) | 0.374 |  |  |
| No | 1.00 (Ref.) | 0.673 |  |  |
| Unknown | 0.00 (0.00- ) | 0.999 |  |  |
| Percutaneous coronary intervention | |  |  |  |
| Yes | 0.66 (0.24-1.80) | 0.414 |  |  |
| No | 1.00 (Ref.) | 0.486 |  |  |
| Unknown | 2.30 (0.23-22.63) | 0.475 |  |  |
| Time from admission to ECMO pump on* | 0.98 (0.95-1.03) | 0.436 |  |  |

*Five data points were missing from admission to ECMO pump onset; thus, the multivariate analysis included 207 participants.

ECPR, extracorporeal cardiopulmonary resuscitation; OR, odds ratio; CI, confidence interval; CPR, cardiopulmonary resuscitation; EMS, emergency medical service; ROSC, return of spontaneous circulation; VF, ventricular fibrillation; VT, ventricular tachycardia; PEA, pulseless electrical activity; ECMO, extracorporeal membrane oxygenation; Ref., reference.
